# Depressive symptom as a risk factor for cirrhosis in patients with primary biliary cholangitis: Analysis based on Lasso‐logistic regression and decision tree models

**DOI:** 10.1002/brb3.3639

**Published:** 2024-08-05

**Authors:** Simin Zhou, Jiwen Li, Jiangpeng Liu, Shijing Dong, Nian Chen, Ying Ran, Haifeng Liu, Xiaoyi Wang, Hui Yang, Man Liu, Hongyu Chu, Bangmao Wang, Yanni Li, Liping Guo, Lu Zhou

**Affiliations:** ^1^ Department of Gastroenterology and Hepatology, General Hospital Tianjin Medical University Tianjin China

**Keywords:** cirrhosis, depressive symptoms, human leukocyte antigen, primary biliary cholangitis

## Abstract

**Background:**

Depressive symptoms are frequently observed in patients with primary biliary cholangitis (PBC). The role of depressive symptoms on cirrhosis has not been fully noticed in PBC. We aimed to establish a risk model for cirrhosis that took depressive symptoms into account.

**Methods:**

Depressive symptoms were assessed by the 17‐item Hamilton Depression Rating Scale (HAMD‐17). HAMD‐17 score was analyzed in relation to clinical parameters. Least absolute shrinkage and selection operator (Lasso)‐logistic regression and decision tree models were used to explore the effect of depressive symptoms on cirrhosis.

**Results:**

The rate of depressive symptom in patients with PBC (*n* = 162) was higher than in healthy controls (*n* = 180) (52.5% vs. 16.1%; *p *< .001). HAMD‐17 score was negatively associated with C4 levels and positively associated with levels of alkaline phosphatase (ALP), γ‐glutamyl transpeptidase (GGT), total bilirubin (TB), Immunoglobulin (Ig) G, and IgM (*r* = −0.162, 0.197, 0.355, 0.203, 0.182, 0.314, *p *< .05). In Lasso‐logistic regression analysis, HAMD‐17 score, human leukocyte antigen (HLA)‐DRB1*03:01 allele, age, ALP levels, and IgM levels (odds ratio [OR] = 1.087, 7.353, 1.075, 1.009, 1.005; *p *< 0.05) were independent risk factors for cirrhosis. Elevated HAMD‐17 score was also a discriminating factor for high risk of cirrhosis in patients with PBC in decision tree model.

**Conclusions:**

Depressive symptoms were associated with disease severity. Elevated HAMD‐17 score was a risk factor for cirrhosis in patients with PBC.

## Graphical abstract

This study demonstrated a high rate of depressive symptoms in patients with PBC. HAMD‐17 score was negatively associated with C4 levels and positively associated with levels of ALP, GGT, TB, IgG, and IgM. Elevated HAMD‐17 score was a discriminating factor for high risk of cirrhosis in Lasso‐logistic regression and decision tree models.

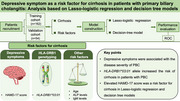

## 1 INTRODUCTION

Primary biliary cholangitis (PBC) is an immune‐mediated cholestatic liver disease characterized by progressive destruction of the small intrahepatic bile ducts (Cordell et al., 2021). Approximately 30%–40% of patients with PBC exhibit an inadequate response to ursodeoxycholic acid (UDCA), the first‐line therapy for PBC, and are at risk of progressing to liver cirrhosis and even liver failure (Pares et al., 2006). Symptom burdens, including depression, anxiety, inability to sleep, pruritus and fatigue, have a large impact on body health in patients with PBC (Mells et al., 2013; Sivakumar & Kowdley, 2021).

Depression, one of the leading causes of disability worldwide, always coexists with many chronic liver diseases (Barboza et al., 2016; Kim et al., 2019; Labenz et al., 2020; Schramm et al., 2014; Sockalingam et al., 2012). Previous studies demonstrated that depressive symptoms were frequently observed in patients with PBC (Biagini et al., 2008; Cauch‐Dudek et al., 1998; Huet et al., 2000; van Os et al., 2007), yet they were still under‐recognized and not routinely screened for. Patients with PBC were commonly prescribed antidepressants to help manage their symptom burdens (Shaheen et al., 2018; Sivakumar & Kowdley, 2021). Depression was demonstrated to be correlated with poor clinical outcomes in chronic liver diseases (Kronsten et al., 2022; Mullish et al., 2014). In patients with autoimmune hepatitis (AIH), depression was one of the key factors responsible for the increased risk of nonadherence to therapy and relapse (Sockalingam et al., 2012). In nonalcoholic fatty liver disease, depression was related to histological severity (Tomeno et al., 2015; Youssef et al., 2013) and poor therapeutic efficacy (Tomeno et al., 2015). A diagnosis of depression before liver transplantation was related to reduced survival after transplantation (Rogal et al., 2016). However, few studies have addressed the effect of depressive symptoms on disease progression and clinical outcomes in patients with PBC.

It was deemed that 40% of patients with PBC would progress to cirrhosis within 10 years, at which point patients were more susceptible to develop liver failure and hepatocellular carcinoma (Harms et al., 2020). Studies have emphasized the higher prevalence of depressive symptoms in cirrhotic patients (18%–58%) (Buganza‐Torio et al., 2019; Mullish et al., 2014; Nardelli et al., 2013) as compared to the general population (10%) (Global Burden of Disease Study 2013 Collaborators, 2015). Depression was related to the substantial morbidity (Buganza‐Torio et al., 2019) and the severity of liver disease (Bianchi et al., 2005) in cirrhosis. Remarkably, depression could influence the progression, treatment adherence, and overall prognosis in cirrhotic patients (Yang et al., 2024). Depression was also an independent predictor of mortality in cirrhosis (Buganza‐Torio et al., 2019; Kronsten et al., 2022; Mullish et al., 2014). Hence, it is of vital importance to incorporate the assessment of depressive symptoms into the management of cirrhosis in patients with PBC.

Previous studies demonstrated that human leukocyte antigen (HLA)‐DRB1 alleles were associated with susceptibility to PBC (Hu et al., 2014; Invernizzi et al., 2008; Li et al., 2022; Wang et al., 2019). HLA allele was related to predisposition risk, staging, symptomatic state, AIH, and hepatocellular carcinoma events in patients with PBC (Khor et al., 2023). HLA‐DRB1 polymorphisms also showed the associations with production of autoantibodies in PBC. HLA‐DRB1*08 predisposed patients with PBC to anti‐mitochondrial antibody (AMA) production (Stone et al., 2002), while DRB1*01 was protective against the incidence of AMA (Li et al., 2022). DRB1*03 and DRB1*16 were risk indicators associated with the incidence of anti‐sp100 antibodies (Li et al., 2022). In addition, DRB1*04:05 and DRB1*08:03 predisposed patients with PBC to anti‐gp210 and anti‐centromere antibodies production, respectively (Nakamura et al., 2010). In addition to the production of autoantibodies, HLA‐DRB1 alleles were also associated with cirrhosis in patient with PBC (Umemura et al., 2012; Wang et al., 2019). In other chronic liver diseases, such as AIH (Czaja, 2009; Czaja et al., 1999; Ma et al., 2021; Montano‐Loza et al., 2006; Muratori et al., 2009), autoimmune sclerosing cholangitis (ASC) (Ma et al., 2021), portal fibrosis (Varma et al., 2016), chronic hepatitis B (Doganay et al., 2014; Han et al., 2012), and chronic hepatitis C (Aikawa et al., 1996; Hue et al., 2002; Urabe et al., 2013), HLA‐DRB1 alleles also conferred a risk or protective effect on cirrhosis. The HLA molecules were appreciated to be associated with the disease susceptibility of not only chronic liver diseases, but also various mental diseases. DRB1*11:01, previously demonstrated to be related to a reduced risk of developing PBC (Chow et al., 2013; Invernizzi et al., 2012; Invernizzi et al., 2008), provided a protective effect on post‐traumatic stress disorder (Katrinli et al., 2019), bipolar affective disorders (Le Clerc et al., 2021), affective distress profile (Vica et al., 2022), and depression of early undifferentiated arthritis (Bandinelli et al., 2021). In the female participants of the UK Biobank cohort, HLA‐DRB1*14:15 and HLA‐C*14:03 were identified to be associated with higher depression score (Cheng et al., 2024). Hence, HLA‐DRB1 alleles should be highly valued in patients with PBC.

In this study, we evaluated the influence of depressive symptoms on disease severity in patients with PBC who received UDCA monotherapy for more than 1 year but less than 2 years. In addition, we investigated the potential association between HLA‐DRB1 alleles and cirrhosis. Further, by employing least absolute shrinkage and selection operator (Lasso)‐logistic regression analysis for feature selection, as well as classification and regression trees (CART) analysis for visualization of risk stratification process, we established a reliable risk model for cirrhosis which took depressive symptoms and HLA‐DRB1 alleles into consideration. It is likely to provide the necessity of extensive screening for depressive symptoms in patients with PBC.

## 2 METHODS

### 2.1 Study participants

Consecutive patients with a diagnosis of PBC, who visited our hepatology clinics from December 2017 to December 2020, were enrolled as a training cohort in the study (*n* = 162). In addition, 54 patients with PBC admitted from January 2021 to January 2023 were recruited as a validation group. To exclude the influence of illness duration on depressive symptoms, patients who received UDCA monotherapy for more than 1 year but less than 2 years were considered for participation. The diagnosis of PBC was based on the 2018 Practice Guidance from the American Association for the Study of Liver Diseases (Lindor et al., 2019). Inclusion criteria were as follows: (1) a diagnosis of PBC, (2) age > 18 years, (3) UDCA monotherapy for more than 1 year but less than 2 years, (4) willingness to participate in structured psychiatric interviews by two trained physicians with the 17‐item Hamilton Depression Rating Scale (HAMD‐17), and (5) willingness to be recruited for HLA‐DRB1 genotyping. Exclusion criteria were as follows: (1) with a severe psychiatric disorder (other than depression); (2) with extrahepatic autoimmune diseases, viral hepatitis, or malignant tumor comorbidities; and (3) incomplete clinical information. Gender‐ and age‐matched individuals from the health management center (*n* = 180) were included as a healthy control group. Characteristics of patients and healthy controls are summarized in Table S1. Written consent was given in writing by all subjects. The study was approved by the Ethics Committee of Tianjin Medical University in accordance with the Declarations of Helsinki (Ethical No. IRB2023‐WZ‐059).

### 2.2 Data collection

HAMD‐17 was used to assess the presence and severity of depressive symptoms in patients who suffered from PBC and received UDCA monotherapy for 1–2 years (Petrak et al., 2015). At the time of the questionnaire, the liver pathology, diagnostic imaging reports, and laboratory parameters were collected. Cirrhosis was diagnosed on the basis of liver pathology. Biopsy confirmation was not necessary when obvious signs of cirrhosis, such as ascites, coagulopathy, and a shrunken nodular appearing liver, were present (Schuppan & Afdhal, 2008). Response to UDCA treatment was evaluated by Paris‐I criteria (Pares et al., 2006). The serum levels of alanine aminotransferase (ALT), aspartate aminotransferase (AST), alkaline phosphatase (ALP), γ‐glutamyl transpeptidase (GGT), and total bilirubin (TB) were determined by an automatic biochemical analyzer (Chemray‐120, Rayto Life and Analytical Sciences Company). Immunoglobulin (Ig) G, IgM, complement component 3 (C3), and C4 were detected by Lumiray‐1600 (Chemray‐120, Rayto Life and Analytical Sciences Company).

### 2.3 Human leukocyte antigen genotyping

All patients with PBC were recruited for HLA‐DRB1 genotyping. The genomic DNA was isolated from 5 mL of peripheral blood by using the DNA extraction kit (DP349, Qiagen). After the amplification of the second and third exons of HLA‐DRB1, the genotyping of HLA‐DRB1 was conducted from polymerase chain reaction with sequence‐based typing by ABI 3500 Genetic Analyzer.

### 2.4 Construction of Lasso‐logistic regression model

In order to select cirrhosis‐related variables for further analysis, Lasso‐logistic regression algorithm was applied in the training cohort. Lasso model was identified as a high‐performing model, which penalized the model coefficients for overoptimism based on the value of lambda (Liang et al., 2020). By imposing a penalty on the absolute size of the regression coefficients, Lasso shrank less important factors toward zero, resulting in a sparse model with only the strongest predictors (Huang et al., 2016; Liang et al., 2020; Okada et al., 2022). The most appropriate tuning parameter lambda was 0.015 when the binomial deviance was minimized. Nine variables with nonzero coefficients were retained in the Lasso analysis (Figure 2a,b). Subsequently, the above nine variables identified by Lasso regression analysis were further selected for the construction of multivariate logistic regression model. The five retained variables which showed statistical significance in the multivariate logistic regression model (*p* < .05) were used for construction of nomogram model. The discrimination ability of the multivariate logistic model was evaluated by calculating the area under the receiver operating characteristic curve (AUC‐ROC) and goodness‐of‐fit statistics.

### 2.5 Construction of CART model

We trained CART model with all the 13 variables (age, gender, HAMD‐17 score, DRB1*03:01 allele, levels of ALT, AST, ALP, GGT, TB, IgG, IgM, C3, and C4). The CART model was developed using a Gini impurity index algorithm in order to determine an optimal split. The best split point was that which minimized the Gini impurity index to increase the purity of the dataset. The root node, the initial partition in the data indicated by greatest mean decreases in the Gini index, was situated at the top of the tree and followed by successive internal nodes. The power of CART model was calculated in the training cohort and validation cohort. The performance of the created model was evaluated by the criteria such as AUC‐ROC, sensitivity, and specificity.

### 2.6 Statistical analysis

SPSS version 26.0 was used in statistical analysis. Continuous variables were expressed as mean ± standard deviation (SD) (data were normally distributed) or median with interquartile range (IQR) (data were non‐normally distributed). An unpaired Student's *t* test (normally‐distributed data) or Mann–Whitney U test (non‐normally distributed data) was conducted to compare continuous variables between two groups. Differences in categorical variables were determined with chi‐square test as appropriate. Lasso‐logistic regression, nomogram, and CART models were established by R software (version 3.4.3.). The rest of the figures were plotted by GraphPad Prism version 9.0. All reported *p*‐values were two‐sided. Statistical significance was determined at *p *< .05.

## 3 RESULTS

### 3.1 Depressive symptoms were correlated with disease severity in patients with PBC who received UDCA monotherapy

Our study assessed the frequency of depressive symptoms in PBC patients who received UDCA monotherapy for more than 1 year but less than 2 years and healthy controls. The frequency of depressive symptoms in patients with PBC was more than three times as high as in the general population (52.5% vs. 16.1%; *p *< .001) (Table 1). Patients with PBC exhibited higher HAMD‐17 score than controls (*p <* .001). In addition, the five factors of HAMD‐17 criteria, including sleep disorder, retardation, cognitive dysfunction, anxiety/somatization, and weight, also reached statistical significance between the above two groups (*p *< .001).

**TABLE 1:** Depressive symptoms in patients with primary biliary cholangitis (PBC) as compared to healthy controls.

	Control (*n* = 180)	PBC (*n* = 162)	*p*‐value
**Five factors of HAMD‐17 (median with IQR)**			
Sleep disorder	1.0 (0.0–2.0)	2.0 (0.0–3.3)	<.001^a^
Retardation	0.0 (0.0–1.0)	2.0 (0.0–4.0)	<.001^a^
Cognitive dysfunction	0.0 (0.0–0.0)	1.0 (0.0–2.0)	<.001^a^
Anxiety/somatization	1.0 (0.0–3.0)	2.5 (1.0–7.0)	<.001^a^
Weight	0.0 (0.0–0.0)	0.0 (0.0–2.0)	<.001^a^
**Depressive symptoms assessment**			
HAMD‐17 score (median with IQR)	3.0 (1.0–5.8)	8.0 (4.0–15.0)	<.001^a^
Depressive symptoms (*n*, %)	29 (16.1%)	85 (52.5%)	<.001^b^

*Note*: Sleep disorder: items 4–6; retardation: items 1, 7, 8, 14; cognitive dysfunction: items 2, 3, 9; anxiety/somatization: items 10–12, 15, 17; weight: item 16. Without depressive symptoms: HAMD‐17 score < 8; with depressive symptoms: HAMD‐17 score ≥ 8.

Abbreviations: HAMD‐17, 17‐item Hamilton Depression Rating Scale; IQR, interquartile range.

^a^ The statistic value was based on Mann–Whitney U test.

^b^ The statistic value was based on chi‐square test.

**FIGURE 1:**
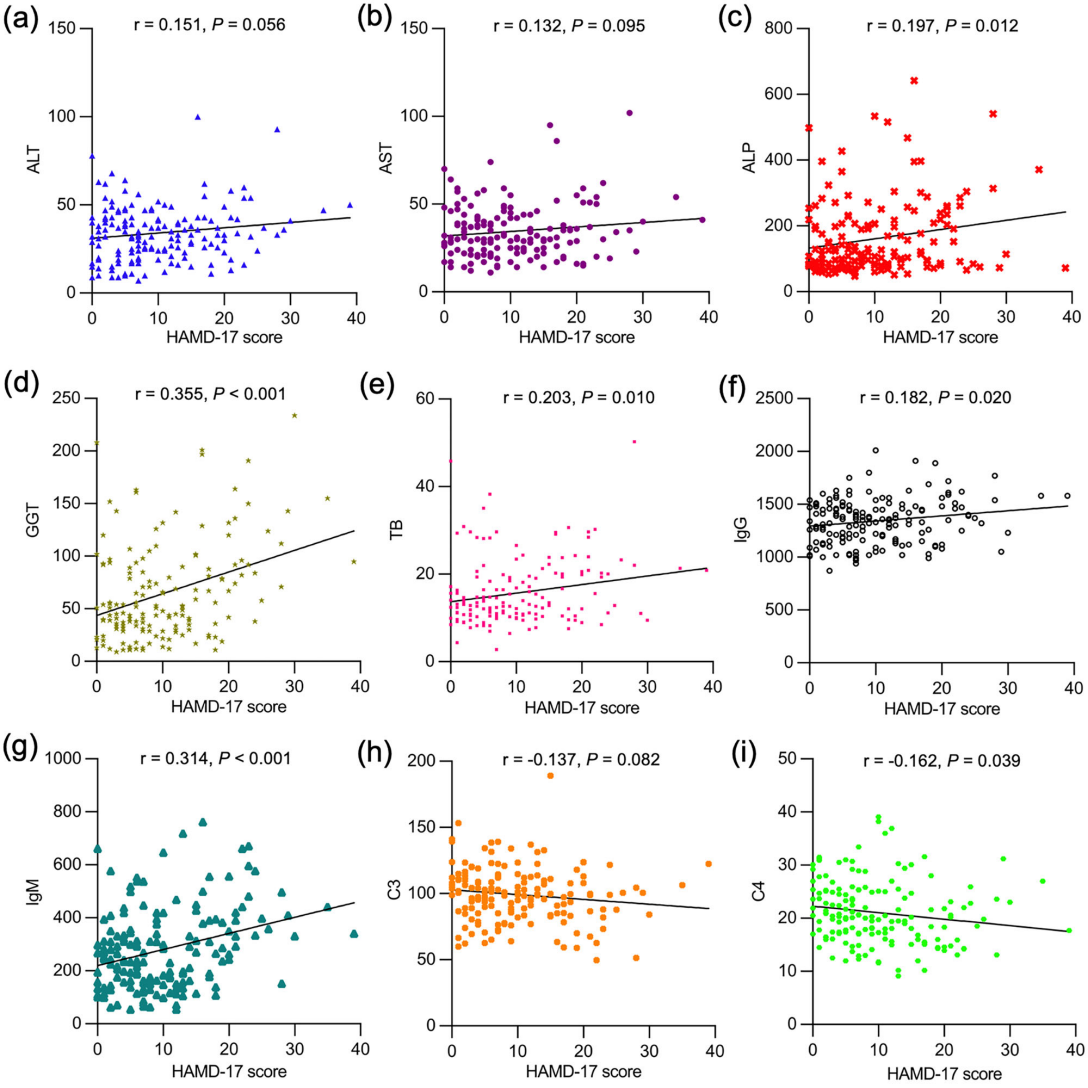
The correlation between 17‐item Hamilton Depression Rating Scale (HAMD‐17) score and laboratory parameters. The Pearson correlation coefficient was calculated to explore the association between HAMD‐17 score and levels of alanine aminotransferase (ALT) (a), aspartate aminotransferase (AST) (b), alkaline phosphatase (ALP) (c), γ‐glutamyl transpeptidase (GGT) (d), total bilirubin (TB) (e), immunoglobulin G (IgG) (f), immunoglobulin M (IgM) (g), complement component 3 (C3) (h), and complement component 4 (C4) (i).

Patients with PBC were divided into two groups: PBC with depressive symptoms group (*n* = 85) and PBC without depressive symptoms group (*n* = 77). Compared to PBC patients without depressive symptoms, patients with depressive symptoms demonstrated poorer response to UDCA treatment (79.2% vs. 55.3%; *p* = .001). The levels of ALP, GGT, and IgM were statistically higher in PBC patients with depressive symptoms than those without depressive symptoms (*p* = .022, .006, .049) (Table S2). In addition, levels of C4 reached statistical significance between the above two groups (*p* = .19). Furthermore, HAMD‐17 score was found to be negatively associated with C4 levels (*r* = −0.162; *p* = .039) and positively associated with levels of ALP (*r* = 0.197; *p* = .12), GGT (*r* = 0.355; *p *< .001), TB (*r* = 0.203; *p* = .010), IgG (*r* = 0.182; *p* = .020), and IgM (*r* = 0.314; *p *< .001) (Figure 1a–i).

### 3.2 DRB1*03:01 allele which was associated with depressive symptoms in patients with PBC increased the risk of cirrhosis

The frequency of HLA‐DRB1 alleles in PBC patients with depressive symptoms was compared with those without depressive symptoms (Table S3). Our study showed that HLA‐DRB1*03:01 allele (odds ratio [OR], 4.253; 95% confidence interval [CI], 1.695–10.671; *p* = .001) was a risk indicator associated with depressive symptoms in patients with PBC. On the contrary, HLA‐DRB1*11:01 allele provided a protective effect against depressive symptoms (OR, 0.166; 95% CI, 0.047–0.587; *p* = .002). Next, we explored the effects of HLA‐DRB1 alleles which were associated with depressive symptoms on cirrhosis in patients with PBC (Table S4). There was no association observed for DRB1*11:01 allele and cirrhosis (OR, 0.686; 95% CI, 0.220–2.140; *p* = .516). Despite the low frequency, DRB1*03:01 allele increased the risk of cirrhosis in patients with PBC (OR, 6.329; 95% CI, 2.849–14.060; *p *< .001).

### 3.3 Elevated HAMD‐17 score was a risk factor for cirrhosis in Lasso‐logistic regression model

**FIGURE 2:**
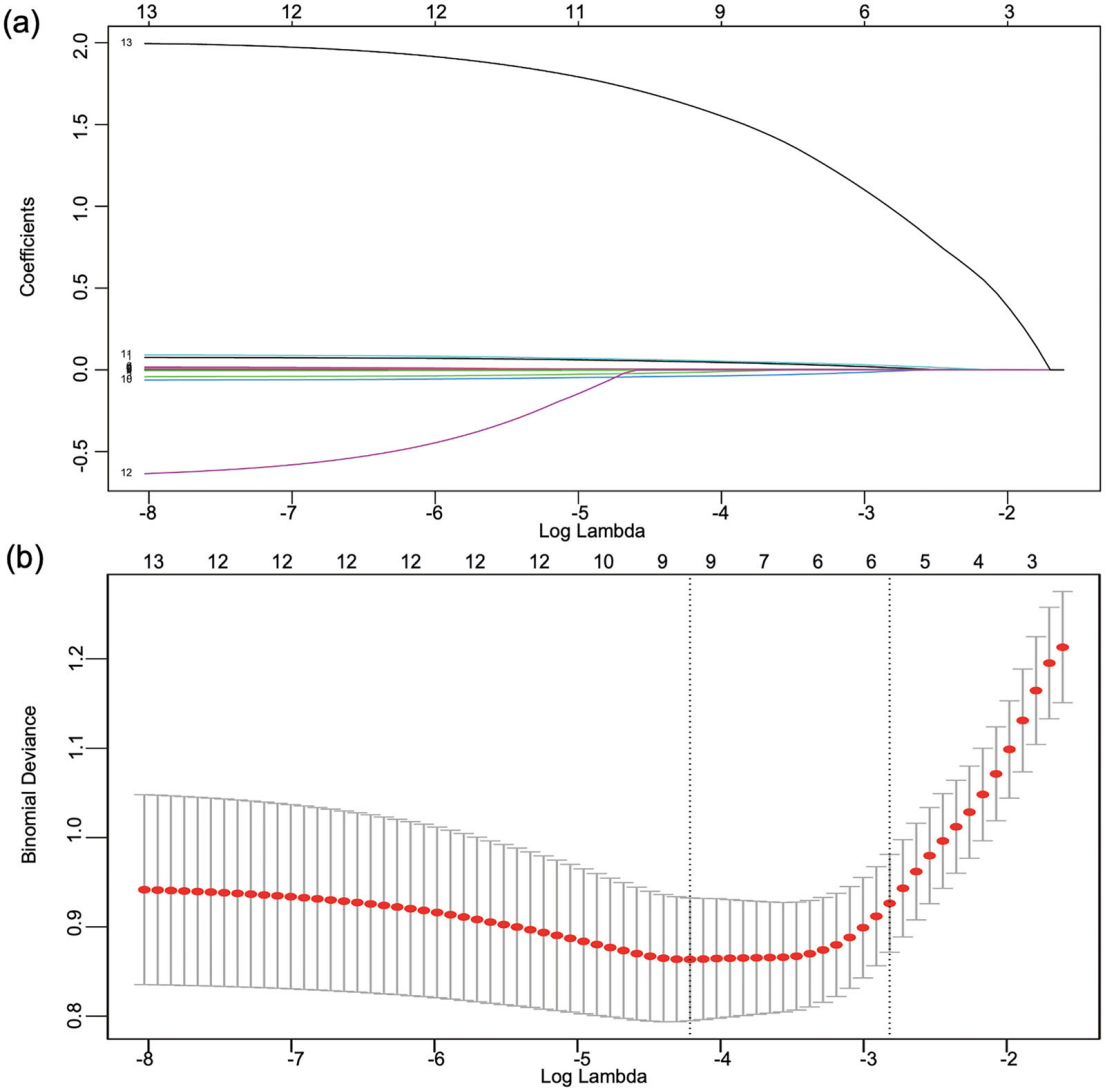
Variable selection using least absolute shrinkage and selection operator (Lasso)‐logistic regression. (a) A coefficient profile plot was produced against the log (lambda) sequence. (b) Nine variables with nonzero coefficients were selected by optimal lambda. By verifying the optimal parameter (lambda), the binomial deviance curve was plotted versus log (lambda). The most appropriate tuning parameter lambda was 0.015 when the binomial deviance was minimized. Dotted vertical lines were drawn based on 1 standard error criteria.

**FIGURE 3:**
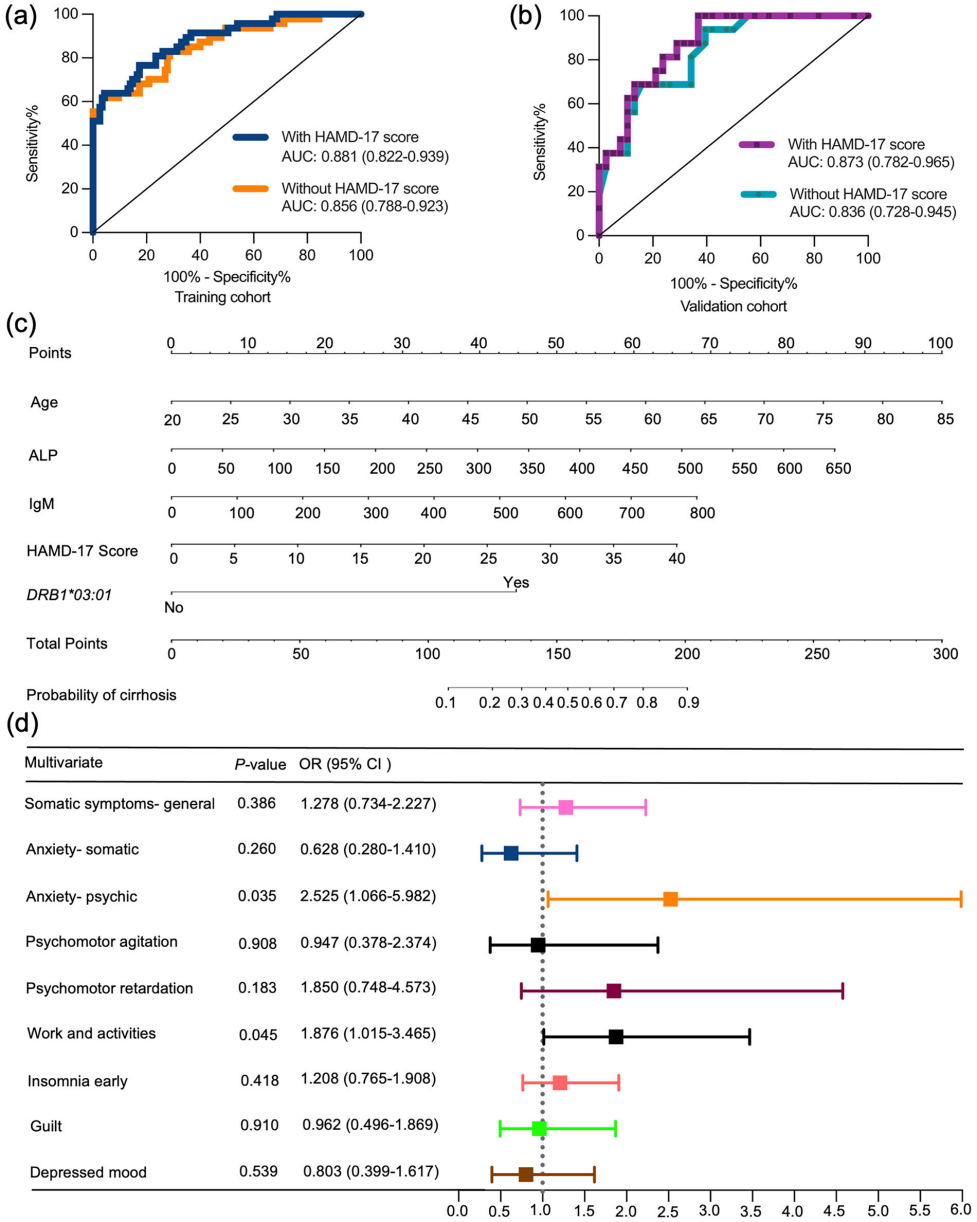
Elevated 17‐item Hamilton Depression Rating Scale (HAMD‐17) score was a risk factor for cirrhosis in logistic regression model. The discrimination ability of the logistic regression model in training cohort (a) and validation cohort (b) was evaluated by calculating the area under the receiver operating characteristic curve (AUC‐ROC). With depression: HAMD‐17 score, HLA‐DRB1*03:01 allele, age, levels of alkaline phosphatase (ALP), and immunoglobulin M (IgM); Without depression: HLA‐DRB1*03:01 allele, age, levels of ALP, and IgM. (c) Nomogram was constructed to identify the expected probability of cirrhosis specific to each patient. (d) Forest plots to explore risk factors for cirrhosis in sub‐items of the HAMD‐17 questionnaire by multivariate logistic regression analysis. Horizontal lines represented 95% confidence interval (CI). The positions of each square demonstrated the odds ratio (OR) point estimate.

We next investigated the risk factors associated with cirrhosis in patients with PBC. Patients with PBC were classified into two groups: with cirrhosis group (*n* = 47) and without cirrhosis group (*n* = 115). In the Lasso regression model, nine out of 13 variables (age, HAMD‐17 score, DRB1*03:01 allele, and levels of AST, ALP, IgG, IgM, C3, and C4) were selected by optimal lambda and further used to build a multivariate logistic regression model (Figure 2a,b). HAMD‐17 score (OR, 1.087; 95% CI, 1.021–1.157; *p* = .009), DRB1*03:01 allele (OR, 7.353; 95% CI, 2.271–23.809; *p *= .001), age (OR, 1.075; 95% CI, 1.028–1.124; *p* = .001), ALP levels (OR, 1.009; 95% CI, 1.003–1.015; *p* = .004), and IgM levels (OR, 1.005; 95% CI, 1.001–1.009; *p* = .020) were independent risk factors for cirrhosis in the multivariate logistic regression model (Table 2). The final model included the above five indicators reached an AUC of 0.881 (95% CI, 0.822–0.939; *p* < .001) and goodness‐of‐fit *p*‐value of .540. What is more, the removal of the HAMD‐17 score variable decreased the model performance, with an AUC of 0.856 (95% CI, 0.788–0.923; *p *< .001) and goodness‐of‐fit *p*‐value of .092 (Figure 3a). In the validation cohort, the model performance reached an AUC of 0.873 (95% CI, 0.782–0.965; *p* < 0.001) with the variable of HAMD‐17 score, and 0.836 (95% CI, 0.728–0.945; *p *< .001) without the variable of HAMD‐17 score (Figure 3b). Finally, to identify the expected probability of cirrhosis specific to each patient, a nomogram model was constructed using the above five indicators (Figure 3c).

**TABLE 2:** Multivariate regression analysis investigating factors associated with cirrhosis in patients with primary biliary cholangitis (PBC).

Variable	Multivariate	
	OR (95% CI)	*p‐*value
Age (years)	1.075 (1.028–1.124)	.001^**^
HAMD‐17 score	1.087 (1.021–1.157)	.009^**^
DRB1*03:01 allele	7.353 (2.271–23.809)	.001^**^
AST (U/L)	0.961 (0.921–1.002)	.064
ALP (U/L)	1.009 (1.003–1.015)	.004^**^
IgG (mg/dL)	0.999 (0.996–1.001)	.206
IgM (mg/dL)	1.005 (1.001–1.009)	.020^*^
C3 (mg/dL)	0.994 (0.968–1.021)	.660
C4 (mg/dL)	0.947 (0.863–1.040)	.257

Abbreviations: ALP, alkaline phosphatase; AST, aspartate aminotransferase; C3, complement component 3; C4, complement component 4; CI, confidence interval; HAMD‐17, 17‐item Hamilton Depression Rating Scale; Ig, immunoglobulin; OR, odds ratio.

* *p* < 0.05.

** *p* < 0.01.

### 3.4 The scores of item “work and activities” and item “anxiety‐ psychic” of the HAMD‐17 criteria were risk factors for cirrhosis

Cirrhosis was more frequent in PBC patients with depressive symptoms than in those without depressive symptoms (44.7% vs. 11.7%; *p *< .001) (Table S2). Next, we explored the association between scores of each item and cirrhosis to distinguish high risk factors for cirrhosis in sub‐items of the HAMD‐17 criteria. By univariate logistic regression, nine out of 17 items (items 1, 2, 4, 7, 8, 9, 10, 11, and 13) were associated with cirrhosis (all *p *< .05) and further used to build a multivariate logistic regression model (Table S5). Item 7 (“work and activities”) (OR, 1.876; 95% CI, 1.015–3.465; *p *= .045) and item 10 (“anxiety‐ psychic”) (OR, 2.525; 95% CI, 1.066–5.982; *p *= .035) showed association with the risk of cirrhosis in multivariate logistic regression model (Figure 3d). There was no multicollinearity in this regression analysis (value of variance inflation factor <10).

### 3.5 Elevated HAMD‐17 score was a risk factor for cirrhosis in CART model

**FIGURE 4:**
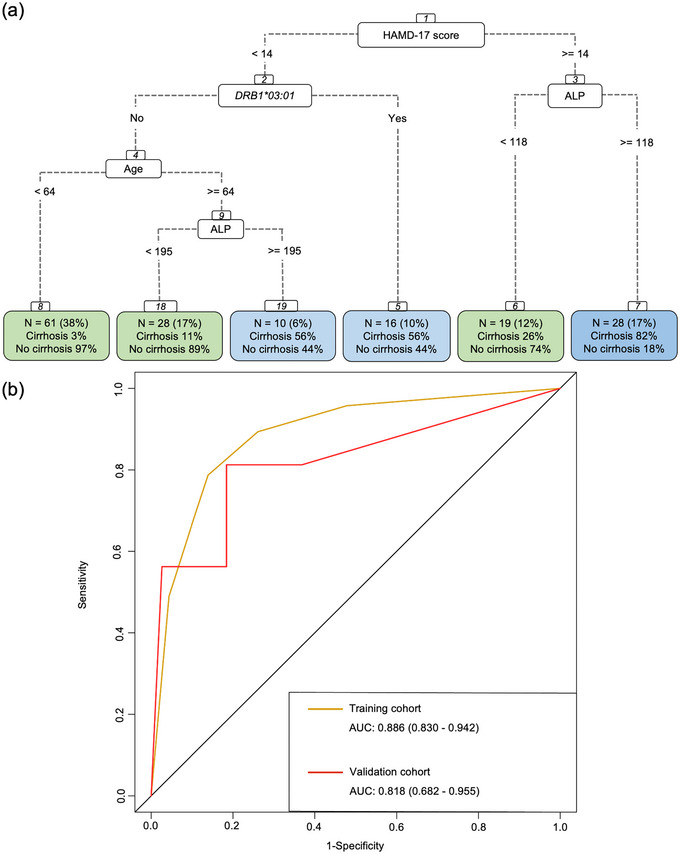
Elevated 17‐item Hamilton Depression Rating Scale (HAMD‐17) score was a discriminating factor for high risk of cirrhosis in classification and regression trees (CART) model. (a) Construction of CART model for visualization. The score of HAMD‐17 served most efficiently as the root node indicated by greatest mean decreases in the Gini index. The decision thresholds were indicated in the branches of the tree. The fraction of appropriately classified patients was seen within each terminal node. The subgroups were marked with green and blue according to prediction outcomes. A blue terminal node indicated categorization as patients with cirrhosis. A green terminal node indicated categorization as patients without cirrhosis. (b) Area under the receiver operating characteristic curve (AUC‐ROC). The CART model worked well in training cohort and validation cohort with an AUC of 0.886 and 0.818. ALP, alkaline phosphatase.

In order to establish a visualized risk model of cirrhosis, we adjusted a CART model with all these 13 variables to find the best combination of indicators and their exact cut‐off values. The fitted decision tree was screened out and shown in Figure 4a. The training cohort had excellent discrimination (AUC, 0.886; 95% CI, 0.830–0.942; sensitivity of 86.1%; specificity of 78.7%) for the identification of cirrhosis. Similarly, evaluation of the validation cohort also displayed good predictive power (AUC, 0.818; 95% CI, 0.682–0.955; sensitivity of 81.6%; specificity of 81.2%) (Figure 4b). Elevated HAMD‐17 score (≥14), the root node indicated by greatest mean decreases in the Gini impurity index, was a discriminating factor for high risk of cirrhosis, while HLA‐DRB1*03:01 allele, high levels of ALP (≥118 and 195 U/L, respectively), as well as increased age (≥64 years) were also found to be important risk factors for cirrhosis in patients with PBC.

## 4 DISCUSSION

In the present study, we found a high prevalence of depressive symptoms diagnosed by HAMD‐17 criteria in patients with PBC. Further, depressive symptoms and the related HLA‐DRB1*03:01 allele were associated with cirrhosis in patients with PBC. Notably, by employing Lasso‐logistic regression for feature selection and CART analysis for visualization of risk variables, we established a reliable risk model for cirrhosis which took depressive symptoms and HLA‐DRB1 alleles into consideration.

Previous studies demonstrated that 30%−50% of the PBC patients were classified as having depressive symptoms based on self‐reported questionnaires (Biagini et al., 2008; Cauch‐Dudek et al., 1998; Huet et al., 2000; van Os et al., 2007). Comparable to previous studies, the overall prevalence of depressive symptoms was 52.5% by HAMD‐17 criteria in our study. This was substantially higher than the prevalence of 3.7% assessed by the Diagnostic and Statistical Manual IV (DSM‐IV) criteria, a structured psychiatric interview (van Os et al., 2007). The difference might be attributed to fatigue and other somatic symptoms which were assessed in HAMD‐17 but not DSM‐IV criteria. Previous studies indicated that depression was associated with severity of chronic liver diseases (Bianchi et al., 2005; Tomeno et al., 2015; Youssef et al., 2013) and extrahepatic autoimmune diseases (Kochar et al., 2018; Liao et al., 2022; Michelsen et al., 2017; Rekvig et al., 2012). However, there was a lack of data regarding the association between depression and the disease severity in patients with PBC. In this study, we observed that PBC patients with depressive symptoms showed higher serum levels of ALP, GGT, and IgM and lower levels of C4 than those without. Cirrhosis was also more frequent in PBC patients with depressive symptoms. These results highlighted the clinical significance of the identification and management of depressive symptoms in patients with PBC.

Accumulating studies demonstrated that HLA‐DRB1 alleles were critical in determining susceptibility to PBC (Hu et al., 2014; Invernizzi et al., 2008; Li et al., 2022; Wang et al., 2019). Moreover, HLA‐DRB1 alleles might also confer a risk or protective effect on cirrhosis in patients with PBC (Umemura et al., 2012; Wang et al., 2019). In our study, DRB1*03:01 allele, a risk indicator associated with depressive symptoms, increased the risk of cirrhosis in patients with PBC. Consistent with our study, relevant studies indicated that the anti‐sp100‐positive patients with PBC who had an increased frequency of DRB1*03:01 suffered from cirrhosis more frequently compared to anti‐sp100‐negative patients (Mytilinaiou et al., 2012; Tana et al., 2015; Wang et al., 2019). DRB1*03 allele was also a risk indicator associated with cirrhosis in AIH (Czaja, 2009; Czaja et al., 1999; Ma et al., 2021; Montano‐Loza et al., 2006; Montano‐Loza et al., 2012), ASC (Ma et al., 2021), and chronic hepatitis C (Hue et al., 2002). DRB1*03, the principal genetic risk factor in AIH (Strettell et al., 1997), was demonstrated to be associated with poor response to corticosteroid therapy (Czaja, 2009; Czaja et al., 1997) and higher median diagnostic scores (van Gerven et al., 2015). Moreover, cirrhosis was more common in the slow responders who had a higher frequency of DRB1*03 than rapid responders in patients with AIH (Czaja, 2009). Production of relevant autoantibodies might be the possible mechanisms by which DRB1*03 increased the risk of cirrhosis. DRB1*03 was related to the concurrence of antibodies to Ro52 and soluble liver antigen, which independently correlated with progression to cirrhosis in patients with AIH (Montano‐Loza et al., 2012). Our findings indicated the considerable impact of HLA‐DRB1 alleles on the risks of cirrhosis, providing valuable insights into the correlation between genetic predisposition and cirrhosis in patients with PBC.

Depression was one of the most common psychiatric morbidities in patients with cirrhosis (Buganza‐Torio et al., 2019; Mullish et al., 2014; Nardelli et al., 2013). A comorbid diagnosis of depression was associated with increased mortality and poor clinical outcomes in cirrhotic patients (Mullish et al., 2014; Yang et al., 2024). If left untreated, early cirrhosis might eventually progress to end‐stage cirrhosis. For better management of PBC, it was pivotal to establish a risk model for cirrhosis which took depression into account. In our study, HAMD‐17 score, DRB1*03:01 allele, age, ALP levels, and IgM levels were independent risk factors for cirrhosis in Lasso‐logistic regression model. In the CART model, an elevated HAMD‐17 score was also a discriminating factor for high risk of cirrhosis. In order to increase the purity of the dataset and get the better result of splitting, the Gini impurity index of this model should be reduced as much as possible. The HAMD‐17 score was selected as the root node which minimized the Gini index and caused the largest separation among subgroups. For patients with a HAMD‐17 score less than 14, the procedure was continued based on the presence or absence of *DRB1∗0301*. In the absence of *DRB1∗0301*, age became a variable of great importance.

Previous studies provided supportive evidence for the beneficial effects of antidepressants on progression of liver cirrhosis. Patients with PBC were commonly prescribed antidepressants to help manage their symptom burdens (Shaheen et al., 2018; Sivakumar & Kowdley, 2021). Mirtazapine was associated with a decreased risk of decompensated cirrhosis, liver transplant, or death in patients with PBC (Shaheen et al., 2018). In thioacetamide‐induced liver fibrosis, mirtazapine could ameliorate fibrosis by mitigating oxidative stress pathway (El‐Tanbouly et al., 2017). Tricyclic antidepressant was also associated with a decreased risk of developing cirrhosis (Chen et al., 2018). This category of drugs could promote accumulation of ceramide through inhibition of acid ceramidase to regulate collagen production in human hepatic stellate cells (Chen et al., 2017). Amitriptyline could ameliorate hepatic steatosis, fibrosis, and liver damage induced by high‐fat diet (Fucho et al., 2014; Moles et al., 2010). Rolipram, also a phosphodiesterase 4 inhibitor, could decrease the expression of hepatic profibrotic cytokines (Gobejishvili et al., 2013) and attenuate collagen deposition (Elnagdy et al., 2023). Accordingly, antidepressant might emerge as a promising therapeutic candidate for cirrhosis in patients with PBC.

By employing Lasso‐logistic regression analysis for feature selection and CART analysis for visualization of the risk stratification process, we aimed to establish a more reliable risk model for cirrhosis. Lasso regression was applied to minimize the potential collinearity and prevent over‐fitting of variables (Liang et al., 2020). Lasso analysis had some advantages over other regression approaches as it performed both variable selection and coefficient shrinkage via cross‐validation, which resulted in a regression solution with improved interpretability and prediction accuracy. Further, Lasso analysis was better suited than other regression methods when the number of events was low (Lee et al., 2019). In addition to Lasso regression method, our research used a visualized CART model to find the best combination of indicators and their exact cutoff values. CART analysis could produce maximum separation among subgroups and minimum variability within clusters (Mondal et al., 2023). CART analysis had some advantages over traditional regression approaches as it accurately stratified prognostic subgroups by identifying the most significant variables and eliminating nonsignificant ones. In addition, it did not require a specified distribution regarding the outcome variables (Altamirano et al., 2017; Burgel et al., 2017; Chester et al., 2019; Makkouk et al., 2017). CART analysis could be more accurate when the relationship between predictors and outcomes was nonlinear (Chester et al., 2019). Furthermore, it was not restricted by a missing value or a small sample size. The partitioning in CART could be graphically represented easily in the form of a decision tree (Luo et al., 2022), making it particularly suitable for clinicians to pay attention to the most significant factors. The application of Lasso‐logistic and CART analysis allowed us to identify the key risk factors from potential indicators, providing a more interpretable model for cirrhosis in PBC.

Our study had several innovations. First, we explored the prevalence of depressive symptoms in patients with PBC and focused on the relationship between depressive symptoms and treatment response to UDCA monotherapy. Second, we explored the effect of depressive symptoms and HLA‐DRB1 alleles on cirrhosis. Last but not least, by employing Lasso‐logistic and CART methods, we established a reliable risk model for cirrhosis. This approach could facilitate identification of cirrhosis, which would contribute to timely intervention. Our study was the first to take depressive symptoms and HLA‐DRB1 alleles into consideration. However, it was a single‐center study. Future studies should focus on increasing sample size and validating our results in a multicenter cohort. Second, HAMD‐17 was used in this study to explore the presence and severity of depressive symptoms. Patients might have some features of a depression, but might not satisfy all the DSM‐IV criteria which were considered necessary to diagnose a major depression. Third, depressive symptoms were only assessed at a single time point in this study. Longitudinal studies with serial depressive symptoms assessment should be carried out. Fourth, the interaction between cirrhosis and depression was complex and bidirectional. Whether depression was the cause or the outcome of cirrhosis remained obscure, which required more confounding factors to be enrolled and further confirmation in prospective studies.

## 5 CONCLUSIONS

Our study demonstrated a high rate of depressive symptoms in patients with PBC. HAMD‐17 score was negatively associated with C4 levels and positively associated with levels of ALP, GGT, TB, IgG, and IgM. On this basis, our study explored the role of depressive symptoms and HLA‐DRB1 alleles on the progression of cirrhosis in patients with PBC. Elevated HAMD‐17 score was a discriminating factor for high risk of cirrhosis in Lasso‐logistic regression and CART models. The construction of the risk models could facilitate identification of cirrhosis and contribute to timely intervention in patients with PBC. Our findings indicated the considerable impact of depressive symptoms on the risks of cirrhosis, providing valuable insights into the clinical significance of the prompt identification and proper management of depressive symptoms in patients with PBC. It stressed the necessity to incorporate the extensive assessment of depressive symptoms into the clinical management of cirrhosis in PBC.

## AUTHOR CONTRIBUTIONS

**Simin Zhou**: Conceptualization; data curation; formal analysis; visualization; writing—original draft. **Jiwen Li**: Data curation; investigation; software; visualization. **Jiangpeng Liu**: Data curation; formal analysis; visualization. **Shijing Dong**: Conceptualization; writing—review and editing. **Nian Chen**: Conceptualization; writing—review and editing. **Ying Ran**: Methodology. **Haifeng Liu**: Conceptualization. **Xiaoyi Wang**: Methodology. **Hui Yang**: Validation. **Man Liu**: Methodology. **Hongyu Chu**: Methodology. **Bangmao Wang**: Conceptualization; funding acquisition. **Yanni Li**: Data curation; supervision; visualization. **Liping Guo**: Conceptualization; project administration. **Lu Zhou**: Conceptualization; funding acquisition; resources; writing—review and editing.

## CONFLICT OF INTEREST STATEMENT

The authors declare no conflicts of interest.

### PEER REVIEW

The peer review history for this article is available at https://publons.com/publon/10.1002/brb3.3639.

## Supporting information

Supporting Information

## Data Availability

Data are available from the corresponding authors upon reasonable request.
