# Supplementary material for: Depressive symptom as a risk factor for cirrhosis in patients with primary biliary cholangitis: Analysis based on Lasso‐logistic regression and decision tree models
Source: Brain Behav. 2024 Aug 5;14(8):e3639. doi: 10.1002/brb3.3639 (PMC11298689; doi:10.1002/brb3.3639)
Supplement: Supplementary file 1 — Supporting Information [file BRB3-14-e3639-s001.docx]

*To the editor of* Prof. Lu Zhou

*Brain and Behavior*  Department of Gastroenterology and Hepatology,

Tianjin Medical University General Hospital, China

lzhou01@tmu.edu.cn

04 May 2024

**Depressive symptom as a risk factor for cirrhosis in patients with primary biliary cholangitis: analysis based on Lasso-logistic regression and decision tree models**

Simin Zhou^1^ | Jiwen Li^1^ | Jiangpeng Liu^1^ | Shijing Dong^1^ | Nian Chen^1^ | Ying Ran^1^ | Haifeng Liu^1^ | Xiaoyi Wang^1^ | Hui Yang^1^ | Man Liu^1^ | Hongyu Chu^1^ | Bangmao Wang^1^ | Yanni Li^1^ | Liping Guo^1^ | Lu Zhou^1^

^1^Department of Gastroenterology and Hepatology, General Hospital, Tianjin Medical University, No. 154, Anshan Road, Tianjin 300052, China

**Correspondence**

Lu Zhou, Department of Gastroenterology and Hepatology, General Hospital, Tianjin Medical University, Tianjin 300052, China. Email: [lzhou01@tmu.edu.cn](mailto:lzhou01@tmu.edu.cn)

Liping Guo, Department of Gastroenterology and Hepatology, General Hospital, Tianjin Medical University, Tianjin 300052, China. Email: glp0626@163.com

Yanni Li, Department of Gastroenterology and Hepatology, General Hospital, Tianjin Medical University, Tianjin 300052, China. Email: yanni19907300@gmail.com

**TABLE S1** General characteristics, laboratory parameters, and depressive symptoms assessment between patients with PBC and healthy controls.

|  | **Control cohort**  **(n = 180)** | **Model cohort**  **of PBC (n = 162)** | **Validation cohort of PBC (n = 54)** | ***P*_1-_**  **value** | ***P*_2-_value** |
| --- | --- | --- | --- | --- | --- |
| **General characteristics** |  |  |  |  |  |
| Age (median with IQR) | 57.0 (49.0-65.0) | 58.5 (49.0-66.0) | 60.0 (53.5-65.0) | 0.631^†^ | 0.618^†^ |
| Female (n, %) | 150 (83.3%) | 135 (83.3%) | 45 (83.3%) | > 0.99^‡^ | > 0.99^‡^ |
| **Laboratory parameters** |  |  |  |  |  |
| ALT (median with IQR, U/L) | 20.0 (15.0-26.8) | 33.0 (23.0-43.0) | 29.0 (22.0-42.3) | < 0.001^†^ | 0.495^†^ |
| AST (median with IQR, U/L) | 22.0 (16.0-27.0) | 31.0 (24.0-42.0) | 29.0 (22.8-39.0) | < 0.001^†^ | 0.236^†^ |
| ALP (median with IQR, U/L) | 76.0 (64.3-90.0) | 117.0 (80.0-207.0) | 133.0 (99.0-203.0) | < 0.001^†^ | 0.138^†^ |
| GGT (median with IQR, U/L) | 19.0 (13.0-27.8) | 51.0 (31.8-90.5) | 64.0 (33.5-102.8) | < 0.001^†^ | 0.371^†^ |
| TB (median with IQR, μmol/L) | 10.0 (8.4-11.5) | 13.1 (10.3-19.6) | 15.2 (11.9-19.6) | < 0.001^†^ | 0.155^†^ |
| IgG (mean ± SD, mg/dL) |  | 1342.4 ± 210.3 | 1303.3 ± 158.1 |  | 0.212^§^ |
| IgM (median with IQR, mg/dL) |  | 264.0 (157.0-376.8) | 206.5 (127.3-467.5) |  | 0.341^†^ |
| C3 (mean ± SD, mg/dL) |  | 99.2 ± 21.0 | 96.4 ± 27.4 |  | 0.499^§^ |
| C4 (mean ± SD, mg/dL) |  | 21.0 ± 6.0 | 20.4 ± 7.5 |  | 0.548^§^ |
| **Depressive symptoms assessment** |  |  |  |  |  |
| Depressive symptoms (n, %) | 29 (16.1%) | 85 (52.5%) | 28 (51.9%) | < 0.001^†^ | 0.937^‡^ |
| **Disease assessment** |  |  |  |  |  |
| Cirrhosis (n, %) |  | 47 (29.0%) | 16 (29.6%) |  | 0.931^‡^ |

*Note*: Without depressive symptoms: HAMD-17 score < 8; With depressive symptoms: HAMD-17 score >= 8. ^†^: the statistic value was based on Mann-Whitney U test; ^‡^: the statistic value was based on Chi-Square test; ^§^: the statistic value was based on Student’s t test. *P*_1_-value: Training cohort of patients with PBC versus healthy controls; *P*_2_- value: Training cohort of patients with PBC versus validation cohort of patients with PBC.

Abbreviations: PBC, primary biliary cholangitis; IQR, interquartile range; SD, standard deviation; ALT, alanine aminotransferase; AST, aspartate aminotransferase; ALP, alkaline phosphatase; GGT, γ-glutamyl transpeptidase; TB, total bilirubin; Ig, immunoglobulin; C3, complement component 3; C4, complement component 4.

**TABLE S2** Characteristics in PBC-patients with and without depressive symptoms.

|  | **Without depressive symptoms (n = 77)** | **With depressive symptoms (n = 85)** | ***P-*value** |
| --- | --- | --- | --- |
| **General characteristics** |  |  |  |
| Age (mean ± SD) | 57.4 ± 12.3 | 56.7 ± 11.7 | 0.688^§^ |
| Female (n, %) | 61 (79.2%) | 74 (87.1%) | 0.181^‡^ |
| **Laboratory parameters** |  |  |  |
| ALT (median with IQR, U/L) | 32.0 (19.0-43.0) | 33.0 (25.0-42.0) | 0.380^†^ |
| AST (median with IQR, U/L) | 30.0 (23.0-40.0) | 31.0 (24.0-44.0) | 0.725^†^ |
| ALP (median with IQR, U/L) | 107.0 (72.5-182.5) | 127.0 (85.5-242.5) | 0.022^†^ |
| GGT (median with IQR, U/L) | 41.0 (29.0-75.0) | 66.0 (35.5-98.5) | 0.006^†^ |
| TB (median with IQR, μmol/L) | 12.5 (9.9-16.1) | 15.1 (10.7-20.4) | 0.063^†^ |
| IgG (mean ± SD, mg/dL) | 1309.1 ± 198.1 | 1372.6 ± 217.6 | 0.055^§^ |
| IgM (median with IQR, mg/dL) | 237.9 (149.5-312.5) | 293.0 (165.3-404.1) | 0.049^†^ |
| C3 (mean ± SD, mg/dL) | 101.6 ± 20.3 | 97.0 ± 21.4 | 0.164^§^ |
| C4 (median with IQR, mg/dL) | 21.4 (17.4-25.4) | 19.4 (15.5-23.7) | 0.019^†^ |
| **Disease assessment** |  |  |  |
| Cirrhosis (n, %) | 9 (11.7%) | 38 (44.7%) | < 0.001^‡^ |
| UDCA response (n, %) | 61 (79.2%) | 47 (55.3%) | 0.001^‡^ |

*Note*: Without depressive symptoms: HAMD-17 score < 8; With depressive symptoms: HAMD-17 score >= 8. ^†^: the statistic value was based on Mann-Whitney U test; ^‡^: the statistic value was based on Chi-Square test; ^§^: the statistic value was based on Student’s t test.

Abbreviations: IQR, interquartile range; SD, standard deviation; ALT, alanine aminotransferase; AST, aspartate aminotransferase; ALP, alkaline phosphatase; GGT, γ-glutamyl transpeptidase; TB, total bilirubin; Ig, immunoglobulin; C3, complement component 3; C4, complement component 4; UDCA, ursodeoxycholic acid.

**TABLE S3** Association between HLA-DRB1 genotyping and depressive symptoms in patients with PBC.

| ***DRB1*** | **Without depressive symptoms 2n = (154)** | **With depressive symptoms 2n = (170)** | **OR (95% CI)** | ***P-*value** |
| --- | --- | --- | --- | --- |
| *01:01* | 6 (3.9%) | 8 (4.7%) | 1.218 (0.413-3.593) | 0.720 |
| *01:02* | 2 (1.3%) | 2 (1.2%) | 0.905 (0.126-6.502) | > 0.99 |
| *03:01* | 6 (3.9%) | 25 (14.7%) | 4.253 (1.695-10.671) | 0.001^**^ |
| *04:01* | 2 (1.3%) | 1 (0.6%) | 0.450 (0.040-5.009) | 0.931 |
| *04:05* | 4 (2.6%) | 7 (4.1%) | 1.610 (0.462-5.612) | 0.451 |
| *04:06* | 2 (1.3%) | 1 (0.6%) | 0.450 (0.040-5.009) | 0.931 |
| *04:07* | 1 (0.6%) | 1 (0.6%) | 0.905 (0.056-14.599) | > 0.99 |
| *04:10* | 1 (0.6%) | 1 (0.6%) | 0.905 (0.056-14.599) | > 0.99 |
| *07:01* | 27 (17.5%) | 28 (16.5%) | 0.927 (0.519-1.657) | 0.799 |
| *08:01* | 2 (1.3%) | 1 (0.6%) | 0.450 (0.040-5.009) | 0.931 |
| *08:03* | 12 (7.8%) | 11 (6.5%) | 0.819 (0.350-1.913) | 0.644 |
| *09:01* | 15 (9.7%) | 20 (11.8%) | 1.236 (0.609-2.508) | 0.558 |
| *10:01* | 4 (2.6%) | 4 (2.4%) | 0.904 (0.222-3.677) | > 0.99 |
| *11:01* | 15 (9.7%) | 3 (1.8%) | 0.166 (0.047-0.587) | 0.002^**^ |
| *12:01* | 3 (1.9%) | 4 (2.4%) | 1.213 (0.267-5.507) | > 0.99 |
| *12:02* | 7 (4.5%) | 7 (4.1%) | 0.902 (0.309-2.632) | 0.850 |
| *13:01* | 4 (2.6%) | 4 (2.4%) | 0.904 (0.222-3.677) | > 0.99 |
| *14:01* | 5 (3.2%) | 4 (2.4%) | 0.718 (0.189-2.724) | 0.880 |
| *14:05* | 6 (3.9%) | 7 (4.1%) | 1.059 (0.348-3.224) | 0.919 |
| *14:07* | 2 (1.3%) | 1 (0.6%) | 0.450 (0.040-5.009) | 0.931 |
| *14:14* | 1 (0.6%) | 1 (0.6%) | 0.905 (0.056-14.599) | > 0.99 |
| *14:54* | 2 (1.3%) | 2 (1.2%) | 0.905 (0.126-6.502) | > 0.99 |
| *15:01* | 17 (11.0%) | 19 (11.2%) | 1.014 (0.507-2.030) | 0.969 |
| *15:02* | 5 (3.2%) | 5 (2.9%) | 0.903 (0.256-3.181) | > 0.99 |
| *16:02* | 3 (1.9%) | 3 (1.8%) | 0.904 (0.180-4.548) | > 0.99 |

*Note*: Without depressive symptoms: HAMD-17 score < 8; With depressive symptoms: HAMD-17 score >= 8. ^**^: *P <* 0.01.

Abbreviations: OR, odds ratio; CI, confidence interval.

**TABLE S4** Association between HLA-DRB1 genotyping and cirrhosis in patients with PBC.

| ***DRB1*** |  | **Univariate** | |
| --- | --- | --- | --- |
|  |  | **OR (95% CI)** | ***P*-value** |
| *03:01* |  | 6.329 (2.849-14.060) | < 0.001^***^ |
| *11:01* |  | 0.686 (0.220-2.140) | 0.516 |

*Note*: ^***^: *P <* 0.001.

Abbreviations: OR, odds ratio; CI, confidence interval.

**TABLE S5** Risk factors for cirrhosis in sub-items of the HAMD-17 scale by univariate logistic regression analysis.

| **Variable** | **Univariate** | |  |
| --- | --- | --- | --- |
|  | **OR (95% CI)** | ***P*-value** |  |
| Depressed mood^1^ | 1.648 (1.173-2.315) | 0.004^**^ |  |
| Guilt^2^ | 1.788 (1.235-2.588) | 0.002^**^ |  |
| Suicide^3^ | 1.482 (0.937-2.344) | 0.092 |  |
| Insomnia early^4^ | 1.629 (1.110-2.391) | 0.013^*^ |  |
| Insomnia middle^5^ | 1.189 (0.813-1.737) | 0.372 |  |
| Insomnia late^6^ | 1.455 (0.988-2.142) | 0.057 |  |
| Work and activities^7^ | 2.603 (1.699-3.989) | < 0.001^***^ |  |
| Psychomotor retardation^8^ | 3.427 (1.704-6.891) | 0.001^**^ |  |
| Psychomotor agitation^9^ | 1.834 (1.009-3.334) | 0.047^*^ |  |
| Anxiety, psychic^10^ | 2.582 (1.732-3.849) | < 0.001^***^ |  |
| Anxiety, somatic^11^ | 1.899 (1.358-2.655) | < 0.001^***^ |  |
| Loss of appetite^12^ | 1.293 (0.856-1.954) | 0.222 |  |
| Somatic symptoms, general^13^ | 2.152 (1.405-3.296) | < 0.001^***^ |  |
| Sexual interest^14^ | 0.634 (0.286-1.406) | 0.262 |  |
| Hypochondriasis^15^ | 1.339 (0.924-1.941) | 0.123 |  |
| Loss of weight^16^ | 1.378 (0.935-2.030) | 0.105 |  |
| Insight^17^ | 1.440 (0.482-4.307) | 0.514 |  |

*Note*: ^*^: *P <* 0.05; ^**^: *P <* 0.01; ^***^: *P <* 0.001.

Abbreviations: OR, odds ratio; CI, confidence interval.
